# A86 INFORMING MODERN CARE FOR CANADIAN IBD PATIENTS: A PROSPECTIVE RANDOMIZED TRIAL ON APPOINTMENT TYPES

**DOI:** 10.1093/jcag/gwad061.086

**Published:** 2024-02-14

**Authors:** C Galts, B Siempelkamp, K Cade, L Wilson, D Loomes

**Affiliations:** McMaster University, Hamilton, ON, Canada; The University of British Columbia, Vancouver, BC, Canada; pacific digestive health, Victoria, BC, Canada; pacific digestive health, Victoria, BC, Canada; The University of British Columbia, Vancouver, BC, Canada

## Abstract

**Background:**

There is now significant data supporting virtual care (telehealth or telephone) as a means to provide care to IBD patients with equivalent outcomes to in-person care. Many studies assessing virtual care lack a control group and suffer from recall bias. Further, there is a lack of understanding individual patient appointment style preferences and the reasons for these preferences.

**Aims:**

We aimed to determine patient’s preferred appointment styles and to inform these preferences by assessing demographic factors and other appointment related factors (e.g. cost, time required, communication, privacy etc). We hypothesize that this information may help to inform decisions regarding choosing the optimal appointment for patients.

**Methods:**

In this single centre randomized trial we assigned IBD patients to in-person, telehealth (with video), or telephone appointments in a sequential manner. To minimize recall bias, surveys were completed after each appointment style. All participants had demographic data collected and survey data was compiled using UBC redcap. Standard regression analyses and T-scores were used for assessment of statistical significance.

**Results:**

After exclusion of surveys with incomplete data, a total of 81 surveys were included, 27 in-person, 28 telephone, and 26 telehealth. The overall scores (out of ten) were 9.1 ±1.0, 7.8 ±2.1, and 8.0 ±2.6 for in-person, telephone and telehealth appointments respectively. With exclusion of telehealth appointments which suffered technical difficulties (n=6) the overall score improved and range was comparable to in-person appointments (8.9 ±1.2). In-person appointments were associated with a higher cost and longer time commitment but had the highest scores across all appointment features (e.g. perceived privacy, physician engagement etc.). Among patients who would have preferred an in-person appointment optimal communication (80.0%) and interaction with care provider (83.3%) were prioritized. Conversely, among participants who would have preferred telehealth appointments, time savings (71.4%) and cost savings (42.9%) were prioritized. Age, gender, number of dependents, and perceived privacy were not associated with any appointment style preference.

**Conclusions:**

This study concludes that all appointment styles have certain benefits and drawbacks that individual patients may variably prioritize. In-person appointments had a higher cost and time requirement but still remained the highest rated appointment style. As virtual care continues to be part of the standard of care for patients with IBD, we suggest that providers individualize the style of patient appointment to their patients and the expected nature of that encounter.

**Funding Agencies:**

None

